# Divergent Trends in Abortion and Birth Control Practices in Belarus, Russia and Ukraine

**DOI:** 10.1371/journal.pone.0049986

**Published:** 2012-11-30

**Authors:** Boris P. Denisov, Victoria I. Sakevich, Aiva Jasilioniene

**Affiliations:** 1 Laboratory of Population Economics and Demography, Moscow State University, Moscow, Russia; 2 Institute of Demography, National Research University Higher School of Economics, Moscow, Russia; 3 Laboratory of Demographic Data, The Max Planck Institute for Demographic Research, Rostock, Germany; Tehran University of Medical Sciences, Iran (Islamic Republic Of)

## Abstract

**Context:**

The last decade witnessed growing differences in abortion dynamics in Belarus, Russia, and Ukraine despite demographic, social, and historical similarities of these nations. This paper investigates changes in birth control practices in the three countries and searches for an explanation of the diverging trends in abortion.

**Methods:**

Official abortion and contraceptive use statistics, provided by national statistical agencies, were analysed. Respective laws and other legal documents were examined and compared between the three countries. To disclose inter-country differences in prevalence of the modern methods of contraception and its association with major demographic and social factors, an analysis of data from national sample surveys was performed, including binary logistic regression.

**Results:**

The growing gap in abortion rate in Belarus, Russia, and Ukraine is a genuine phenomenon, not a statistical artefact. The examination of abortion and prevalence of contraception based on official statistics and three national sample surveys did not reveal any unambiguous factors that could explain differences in abortion dynamics in Belarus, Russia, and Ukraine. However, it is very likely that the cause of the inter-country discrepancies lies in contraceptive behavior itself, in adequacies of contraceptive knowledge and practices. Additionally, large differences in government policies, which are very important in shaping contraceptive practices of the population, were detected.

**Conclusion:**

Since the end of the 1990s, the Russian government switched to archaic ideology in the area of reproductive health and family planning and neglects evidence-based arguments. Such an extreme turn in the governmental position is not observed in Belarus or Ukraine. This is an important factor contributing to the slowdown in the decrease of abortion rates in Russia.

## Introduction

In the former Soviet Union, the three Slavic republics, Belarus, Russia, and Ukraine, were very much alike not only with respect to culture and economic development but also in terms of demographic patterns. In spite of the two decades of independent development, principal demographic characteristics of the three nations are still very similar. In particular, all three countries experience low total fertility rates of 1.3–1.5 and high prevalence of one-child families. Fertility in the three countries was below the replacement level during the last forty years with an exception for a few years in the 1980s. In times of political and economic turmoil of the 1990s, Belarus, Russia, and Ukraine experienced a sharp fertility downturn with a slight recovery in recent years [1], [2], [3], [4].

Deliberate birth control has long been a mass practice in the region. During the Soviet times, one of the most extensively used means of birth control was induced abortion; this is another feature common to all the three countries. The USSR had the world's highest level in induced abortions [5], and its European republics stood out with the highest abortion levels within the USSR [6], [7], [8], [9], [10]. Some researchers named birth-control behavior practiced during the Soviet times as “abortion culture” [11], [12]. Since the beginning of the 1990s, the abortion rates are steadily declining in Belarus, Russia, and Ukraine. However, the speed of this improvement that was nearly equal in all the three countries during the 1990s started to diverge in the early 2000s, showing a steeper progress in Ukraine and especially in Belarus compared to Russia. As a result, the present-day-Russia exhibits substantially higher abortion rates compared to Belarus and Ukraine.

Research on birth control in Belarus, Russia, and Ukraine is scarce and is mostly focused on intra-country developments. Reasons for the recent divergence in abortion rates between the three sister-countries have not been addressed and remain unclear. The aim of this study is to investigate the phenomenon and to provide its possible explanations.

The first hypothesis is that the observed cross-country differences can be attributed to some changes in regulations concerning provision of abortion services and reporting of abortion by providers that could induce changes in data collection systems. We try to find out whether the quality and the completeness of abortion statistics are comparable between the three countries and thus do not permit the possibility that the differences in abortion incidence are a simple statistical artefact. In addition, we look at and compare the country-specific approaches in collecting official data on contraception.

The outcome of pregnancy depends on the desired number and timing of births as well as on knowledge of and accessibility to the means of effective contraception that would allow the realization of reproductive preferences. Abortion is a consequence of unintended pregnancy, which results from contraceptive failure or non- use [13]. Since reproductive preferences and norms of family size of the three populations are similar, and the propensity to terminate unintended pregnancy by abortion is equally high [14], we look for the explanation of the diverging abortion rates in characteristics of contraceptive behavior.

## Data and Methods

This study relies on two types of data: official statistics on abortion and contraceptive use; and survey data that provide micro-level information about contraceptive use and variety of its covariates.

### Official abortion statistics

Abortion statistics are available from several data sources. It appears that figures, which ought to be the same, often are found to be somewhat different. The data sources on abortion are as follows:

- Publications of the national statistical agencies and the national ministries of health (MoHs), in Russia during 2004–2011 it was the Ministry of Health and Social Development.
- UN data provided through the UN Demographic Yearbooks [15]. Annual abortion figures published by the UN correspond well to the national data sources with a few exceptions.
- The WHO European Health for All Database (HFA-DB) [16]. The HFA-DB also contains statistics on abortions, but provided figures are sometimes lower compared to those from the above sources. Differences appear because the WHO uses data supplied by the MoHs, which often include only abortions performed in medical institutions subordinated to the MoHs (see below for more details).
- Online resource “Abortion statistics and other data – Johnston's Archive”, which provides published data from heterogeneous sources [17].

Notable discrepancies in the data coming from different sources are especially characteristic of Ukraine. For example, the number of abortions in Ukraine in 2000 is 345.8 thousand according to the HFA-DB, 408.9 thousand according to the Center for Health Statistics of the Ministry of Health of Ukraine [18], and 434.2 thousand according to the UN Demographic Yearbook [15]. In such cases, we use the data which come from the national statistical agencies as the most reliable.

### Official statistics on contraception

We used the national MoHs' data on the prevalence of two types of contraception: use of intrauterine devices (IUDs) and use of oral contraceptives (hormonal pills). The MoHs do not collect data on using other methods of contraception.

### Nationally representative sample surveys

For the analysis of contraceptive practices and their determinants, we employ survey data (Table 1). In the case of Russia, we used data from the second wave of the Generations and Gender Survey, conducted in 2007 (GGS-Ru-2007). The sampling of GGS-Ru-2007 covers 11,117 respondents aged from 18 to 82 years (each respondent represents one household) from 32 regions of Russia ensuring the survey representativeness on a country scale. Further details about the survey may be found on the website of the Independent Institute for Social Policy (www.socpol.ru). The GGS is a part of the Pan-European Program “Generations and Gender” (see: http://live.unece.org/pau/ggp/Welcome.html). Two waves of the GGS – in 2004 and 2007 – were conducted in Russia by the Independent Institute for Social Policy. At the moment, it is the only source for nationally representative data on family planning in Russia. The GGS questionnaire included a list of contraceptive methods, and respondents, who were younger than 50 and had a partner at the time of the interview, were asked to indicate methods they were currently using to prevent pregnancy. The respective question was: “Are you or your partner/spouse using or doing any of the things listed on the card to prevent pregnancy at this time?”.

**Table 1:** Selected characteristics of women having a partner at the time of the survey, per cent.

	Belarus (N = 4077)	Russia (N = 2032)	Ukraine (N = 4042)
*Age*			
under 25	8.5	10.7	8.7
25–29	15.4	15.9	15.4
30–34	15.8	17.8	16.4
35–39	17.6	16.6	16.9
40–44	21.2	17.8	19.6
45–49	21.6	21.2	22.9
Total	100	100	100
*Mean age*	33.1	35.7	32.5
*Children ever born*			
0	5.8	9.3	11.0
1	32.8	38.9	40.9
2	49.0	42.4	38.9
3+	12.5	9.4	9.1
Total	100	100	100
*Marital status*			
Currently married	93.0	80.6	95.1
Living with a man	7.0	19.4	4.9
Total	100	100	100
*Place of residence*			
rural	33.4	33.9	30.5
urban	51.6	55.5	62.0
capital city	15.0	10.6	7.5
Total	100	100	100
*Education*			
Lower than higher	76.5	71.0	50.5
Higher	23.5	29.0	49.5
Total	100	100	100

Starting in 1995, the UNICEF carries out the Multiple Indicator Cluster Surveys (MICS) aiming to monitor the progress in achieving the millennium goals. The third wave of the MICS, conducted in 2005, included also Belarus and Ukraine. The MICS is a representative sampling survey at the country level. A multi-stage, stratified cluster sampling approach was used for the selection of the survey sample, details can be found on the MICS website (http://www.childinfo.org/mics3_surveys.html). In the case of Belarus, it is the only known survey that provides information about fertility control in this country. Ukraine has other surveys that are devoted to or embrace the issues of reproductive health; the Ukrainian Reproductive Health Survey (1999) [19] and the Ukrainian Demographic and Health Survey (2007) [20] can be mentioned among the most important ones. In order to ensure the comparability of Ukrainian and Belarusian data, we used the Ukrainian MICS data in the present study.

In the MICS, questions about contraception were formulated as follows: “Are you currently doing something or using any method to delay or avoid getting pregnant? Which method are you using?”. These questions were given to all women aged 15 to 49, not necessarily having a partner. Since we sought to make the MICS data as much compatible with the GGS as possible, we did not consider women without a partner at the time of the survey. The GGS and the MICS use somewhat different concepts of partnership. In the GGS, partnership means a stable intimate relationship, regardless whether the partners co-reside or live separately. In the MICS, a partner is supposed to reside with the respondent in the same household. For the sake of comparability, we excluded Russian cases with separate living of partners.

The dependent variable in our analysis of contraceptive behaviour is use of modern contraception. We apply binary logistic regression and present the results in the form of odds ratios. The independent variables include: women's age, the number of children ever born, place of residence, education, and marital status. Age is split by groups: ≤25, 25–29, 30–34, 35–39, 40–45, and 45–49. Categories for the number of children are: 0 (childless), 1, 2, and 3+. Place of residence comprises three categories: rural, urban, and capital city. Due to limited information on education available in the Ukrainian data, we make a distinction only between higher and lower than higher levels of education. Marital status has two categories: registered marriage and cohabitation. We apply a fixed-effects model using a pooled dataset with a country-variable being used as a covariate.

Unfortunately, none of the three surveys includes questions on abortion.

Governmental policies have the potential of suppressing abortion as a birth control method. To find out more about governmental policies in Belarus, Russia, and Ukraine and their role in shaping contraceptive practice in these countries, we examine national legal documents and regulations relevant to the field of reproductive behaviour, reproductive health, and reproductive rights, including those promoting family planning and contraceptive use.

## Trends in Abortion

According to official statistics in 1990, 4.1 million abortions were registered in Russia, 1 million in Ukraine, and 261 thousand in Belarus. Relative indicators of abortion (per 1000 women of reproductive age and per 100 live births) slightly differed between the three countries, but were very high in all of them at that time. The corresponding absolute numbers of abortions in 2010 were: 1.2 million, 177 and 33 thousand. The three countries achieved a significant improvement in reproductive health: in twenty years numbers of abortions dropped by several times. Table 2 shows the decrease in abortions in terms of absolute and relative indicators according to the national statistical agencies.

**Table 2:** Official abortion statistics in Belarus, Russia, and Ukraine.

	1990	1995	2000	2005	2008	2009	2010	2010/1990
Abortions, thousand								
Belarus	261	193	122	65	42	36	33	7.8
Russia	4103	2766	2139	1676	1386	1292	1186	3.5
Ukraine	1019	740	434	264	217	195	177	5.8
Abortions per 1000 women aged 15–49								
Belarus	106.0	74.9	46.2	24.7	16.7	14.4	13.5	7.9
Russia	113.9	72.8	54.2	42.7	36.1	34.2	31.9	3.6
Ukraine	82.6	58.2	34.1	21.3	18.1	16.4	15.1	5.5
Abortions per 100 live births								
Belarus	183	189	129	72	39	33	31	5,9
Russia	206	203	169	121	81	74	67	3.1
Ukraine	155	150	113	62	43	38	36	4.3

*Note: The data include all types of abortions (medical/pharmaceutical, vacuum/mini, and surgical).*

*Data sources*: Statistical Yearbook of The Republic of Belarus: 2011/National Statistical Committee of the Republic of Belarus, Minsk, 2011, p. 228 (in Russian), available: http://belstat.gov.by/homep/ru/publications/archive/2011.php; Demographic Yearbook of Russia 2010: Statistical Handbook/Federal State Statistics Service, Moscow, 2010, p. 172 (in Russian), available: http://www.gks.ru/wps/wcm/connect/rosstat/rosstatsite/main/publishing/catalog/statisticCollections/doc_1137674209312; Statistical Yearbook of Ukraine: 2010/State Statistics Service of Ukraine, 2011, p. 465 (in Ukrainian), available: http://www.ukrstat.gov.ua/.

However, the rate of decline varied by country (Figure S1). Over the period 1990–2010, the average annual rate of decline in the number of abortions per 1000 women aged 15 to 49 constitutes eight per cent in Belarus, seven per cent in Ukraine, and six per cent in Russia. Although in the 1990s the abortion rates were decreasing at the same pace in all the three countries, in the 2000s this parallel trend ceases. In Belarus the annual speed of reduction in abortion rates accelerated to eleven per cent, in Russia it slowed down to five per cent, while in Ukraine it continued at the same annual speed of six per cent. As a result, today Russia continues to maintain one of the highest abortion rates among all countries of the world reporting this kind of statistics [21]. In the meantime, Belarus and Ukraine moved from the top towards the middle of the international ranking; their abortion levels are comparable to those of the United States, England and Wales, Sweden, and France.

## Birth Control Laws and Regulations

### Abortion legislation

In November 1920, Soviet Russia became the first country in the world, which decriminalized and legalized abortions. In 1936, the USSR banned abortions again, allowing them only on ill-health and medical indications. The ban was called off in 1955, and no significant changes were introduced in the relevant USSR legislation since then. Abortion on request was legally permitted up to 12 weeks of gestation, and after 12 weeks it could be performed only for medical reasons. In 1987, the USSR Ministry of Health authorized an abortion during the period up to 28 weeks of pregnancy under a range of non-medical conditions [22], including husband's death during pregnancy, woman's or her husband's imprisonment, a large current family size (more than 5 children), deprivation of parental rights, divorce during pregnancy, pregnancy resulting from rape, and disability of a previously born child.

Abortion laws in Belarus, Russia, and Ukraine did not experience any principal changes in the post-Soviet period. In all of the three countries, abortion procedure and related services are included in the basic package of state-provided health care guarantees. This means that by law, abortion on a woman's request must be performed in a public health care facility free of charge.

In Belarus and Russia, each woman is legally free to choose whether to give birth or not. Woman's right to this decision is defined in Article 27 of the Belarusian law “On health care” enacted in 1993 [23], and in Russia it is stated in Article 36 of the Russian federal law “Fundamentals of the health care of Russian citizens” passed also in 1993 [24]. Both laws assert that abortion can be performed on woman's request up to 12 weeks of gestation or up to 22 weeks of pregnancy in the presence of certain social reasons, and at any stage of pregnancy if there are detrimental medical indications and a woman's consent for abortion. In Belarus, abortion after 12 weeks of pregnancy can take place only in a state-owned (not private) health care institution, whereas in Russia no restrictions exist in this respect. This is the only legislative difference between Belarus and Russia that we could identify. In both Belarus and Russia, the Ministry of Health sanctions the list of medical indications for induced abortion, whereas the Government determines the list of social indications.

Post-Soviet Ukraine confirms woman's right to abortion in the law “Fundamentals of Legislation on Health Care”, Article 50 [25], which went into effect in 1992. According to this law, a woman has a right to request for termination of her pregnancy up to 12 weeks of gestation. Abortion in the period between 12 and 22 weeks of pregnancy is allowed under some specific conditions.

All in all, differences in the regulations defining abortion procedure are really minor in the three countries. Before the 12^th^ week of gestation, abortion is allowed on one and the only condition – a woman's request, and after the 12^th^ week it can be performed only in the presence of medical reasons.

As far as social reasons are concerned, some differences exist between the countries. It is noteworthy, however, that induced abortions performed on the basis of social reasons represent a very small portion of reported abortions (less than 1 per cent), and their contribution to abortion statistics is negligible [26]. Currently, the widest range of social grounds for late abortion is in force in Belarus. It consists of ten items [27]: (1) woman's or her husband's imprisonment; (2) husband's severe disability; (3) having a child with a disability since childhood; (4) husband's death during pregnancy; (5) divorce during pregnancy; (6) decision of a court depriving of parental rights; (7) pregnancy resulted from rape; (8) attained family size (more than three children); (9) woman's or her husband's unemployment or job loss; and (10) woman's refugee status.

Russia used to have a similar list until 2003, when it was dramatically reduced from thirteen to four items [28]: (1) restriction or deprivation of parental rights; (2) woman's imprisonment; (3) husband's disability or his death during pregnancy; (4) rape. In Ukraine, the list of social indications for abortion, analogous to that active in Belarus until now and in Russian prior to 2003, existed until 2006. In 2006, Ukraine entirely removed social indications from the abortion legislation [29]. Before this, in 1996, during “the difficult period of transition”, the Russian government expanded the list of social indications for abortion (Government Decree, May 8, 1996 № 567). The grounds for termination of pregnancy from 12 to 22 weeks could be the following: husband's disability, husband's death during pregnancy, woman's or her husband's imprisonment, woman is not married, unemployment of one or both of the spouses, absence of shelter, family income below the subsistence minimum, court's decision on restriction or deprivation of parental rights, dissolution of marriage during pregnancy, rape, status of refugee or forced migrant, large family size (three children and over), a disabled child. Since 2012 the list of social indications for abortion in Russia contains only one point (rape).

### Access to modern contraception

There are no any special laws regulating use of contraception in the countries under study and thus no legal barriers exist to using it. The only contraceptive method, whose use is subject to some legal regulation, is sterilization. In Russia, sterilization can be performed to a Russian citizen, who is not younger than 35 years of age or has at least two children, upon receiving his/her written request. For medical indications, sterilization is performed irrespective of the person's age or number of children. According to the Belarusian law “On health care” [23], the only condition for obtaining contraceptive sterilization is the legal age of majority (18 years). Ukrainian citizens have no right to get sterilization for contraceptive purposes. Surgical sterilization is permitted in Ukraine only for medical indications, and there are fifteen indications specified for women and three for men [30].

None of the three countries has domestic production of pills. They are all imported, and therefore their prices are relatively high.

Free contraception actions happen episodically during manufacturers' promotion campaigns, anti-AIDS campaigns, and similar events. According to the Ukrainian DHS (2007) data, 6.4 per cent of female respondents had IUDs inserted free of charge, and 1.6 per cent of them had access to free oral contraceptives (pills) [20].

## Official Statistics on Abortions and Contraception: Data Collection Systems

In Belarus, Russia, and Ukraine, most of abortion procedures take place in public health facilities that are ruled by the national MoHs. These ministries are the major abortion-service providers. The national statistical agencies (*Belstat* in Belarus, *Rosstat* in Russia, and *Derzhstat* in Ukraine) collect data on reported abortions from the MoHs and combine them with information obtained from medical institutions of other ministries as well as from private abortion providers. Abortion statistics provided by the statistical agencies are therefore more complete than the data possessed by the MoHs.

In Russia, according to unpublished Rosstat statistics of the 2000s, medical institutions subordinated to the Ministry of Health and Social Development perform about 90 per cent of officially registered abortions, and the private sector contributes another 8–9 per cent. In Belarus and Ukraine, information on the share of abortions performed by private medical facilities and the completeness of respective reporting is ambiguous. The authors of the Strategic Assessment “Abortions and Contraception in Ukraine” [31] assume that some regions in Ukraine do not collect complete data on abortion procedures held in private health care facilities; at the same time they assert that private services are expensive and continue to be unaffordable for the majority of the population. Information about the quality of abortion reporting and registration in Belarus is contradictory. Some experts say [32]: “<…> collection of relevant statistical data is not yet adjusted to meet the development of paid gynaecological services, and part of early pregnancies terminated by vacuum aspiration in private medical centres may miss the registration”. The latter assumption is seemingly based on the observed decline in vacuum aspiration abortions performed in public health facilities in recent years. Other experts believe that the count of abortions performed in the private sector is nearly complete in Belarus [33].

Philipov et al. [34] assessed the reliability and the completeness of Russian abortion statistics by comparing official statistical data on abortions with data from several regional surveys, conducted in 1988/1989, 1996, and 2000. The study focused on abortions performed within two years prior to the survey and found no significant differences between the survey results and official figures. It proves that in spite of growing private medical services, official abortion statistics in Russia are reliable.

Reliability of the official abortion statistics in Ukraine is also supported by surveys. Data from two nationally representative population surveys, conducted in Ukraine in 1999 and 2007, showed quite good matching with officially reported abortion figures. In other words, abortion estimates generated using two different data sources – survey reporting and routine statistical registration – show similar values.

Abortion statistics possessed by the MoH are based on information gathered in specific accounting forms, which are annually filled out by clinics and other medical facilities. The form asks to report not only induced abortions, but also spontaneous (miscarriages), criminal (by definition, criminal abortion is any intervention aiming to terminate pregnancy that takes place outside a special facility authorized to perform an abortion), as well as unspecified abortions (abortions outside a clinic that are not proved to be criminal while the woman did not have her pregnancy registered in a clinic). The extended definition of abortion is the legacy of the USSR data collection. In Russia, as reported by the MHSD, spontaneous abortions accounted for more than 16 per cent of the total number of abortions (using the extended definition) registered in 2010, and unspecified abortions constituted 5 per cent. The respective figures for Ukraine were about 7 and 19 per cent in 2007 [31]. It should be mentioned that during the 1990s this proportion in Ukraine ranged from four to eight per cent [35]. The ban of social indications for later term abortions could have caused a higher proportion of unspecified abortions in 2007 than in the 1990s.

Due to the continued use of the extended definition of abortion, abortion rates in Belarus, Russia and Ukraine are misleadingly inflated and should be used with caution for international comparisons. A proper comparative analysis requires that the above mentioned extensions (e.g., spontaneous and unspecified) are subtracted. The problem is that data on spontaneous and other types of abortions that take place without medical supervision are usually not published. On the other hand, one could argue that the inclusion of these extensions into official abortion statistics compensates possible undercount of abortions performed in the private sector.

To sum up, our analysis shows that the data collection systems in Belarus, Russia and Ukraine are similar. The registered levels of abortion can be considered reliable. The fact that they share similar advantages and suffer from similar deficiencies suggests that official abortion statistics of these countries are of comparable quality and completeness. Consequently, we draw a conclusion that the divergence observed in the post-Soviet abortion trends in these countries is a genuine phenomenon.

Regarding official statistics on contraception, this information in Belarus, Russia and Ukraine is collected via medical networks associated with the MoH only. The MoHs compile data on patients' use of two types of contraception: intrauterine devices (IUDs) and oral contraceptives (hormonal pills). These data can be biased because a variety of pills and other means of contraception are available without doctor's prescription, and there is a growing network of private institutions providing family planning services. Prevalence of contraceptives among patients of public medical networks may therefore differ from corresponding prevalence in the general population. The MoH data are useful, however, as they give an insight into how contraceptive use was changing over time in the three countries.

## Contraceptive Behavior

### Contraceptive use in light of official statistics

Official statistics on contraception collected by the MoHs shows that proportions of women practicing hormonal contraception (pills) were steadily growing in Belarus, Russia, and Ukraine since the early 1990s (Figure S2). Almost throughout the entire period of 1990–2010, the highest level of hormonal contraceptive use was registered in Belarus, which goes well in line with the downward trend observed in abortion rates in this country. Between 1990 and 2010, the proportion of Belarusian women (aged 15–49) using hormonal contraceptives increased from 5 to 20 per cent, with the most rapid increase taking place in the early 2000s. In Ukraine, hormonal contraceptive use was rather low before the 2000s – in the second half of the 1990s it was lower than in Belarus and Russia. A sharp increase in use of pills occurred at the beginning of the 2000s, and in 2009 it reached 19 per cent. Russia remains far behind Belarus and Ukraine, despite the promising increase experienced over the 1990s. In 2010, the level of hormonal contraceptive use in Russia was 12 per cent, which is substantially lower compared to the other two countries.

As regards the prevalence of IUDs, it was among the most commonly used modern contraceptive methods in the late USSR. Its high popularity was a result of the aggressive promotion campaign implemented by the Ministry of Heath of the USSR in the 1980s. After the collapse of the USSR, a variety of contraceptives available on the market has significantly increased, and this method became noticeably less common. In all the three countries under study, the rise in IUD use persisted into the early 1990s, and then went down and levelled off in the 2000s. Throughout the 1990s and the 2000s, Belarus experienced the highest prevalence of IUDs. The trends in IUD use in Russia and Ukraine differed in the 1990s (with Russia showing higher rates of IUD prevalence), but in the 2000s they converged.

IUD and pills are mutually exclusive. If one combines the use of hormonal contraceptives with use of IUDs, one finds that the total contraceptive prevalence increased from 28 to 41 per cent in Belarus and from 18 to 32 per cent in Ukraine over the observed period of 1990–2010, whereas in Russia it has changed only slightly (25 per cent in 2010 versus 19–22 per cent in the early 1990s).

### Contraceptive prevalence as shown by survey data

The data from the national sample surveys, which were carried out at approximately the same time in Belarus, Russia, and Ukraine, show high levels of contraceptive use in all the three countries (Table 3). The highest proportion of women having a partner and using any contraceptive method was found in Russia (77 per cent); Belarus shows a slightly lower indicator (74 per cent), whereas Ukraine holds the last place in this respect (69 per cent). This level of contraceptive prevalence is similar to that typical of western countries such as France, the Netherlands, Germany, Portugal, or USA [36]

**Table 3:** Contraceptive prevalence among women aged 15 to 49 who have a partner, in per cent.

	Belarus, 2005	Russia, 2007	Ukraine, 2005
Any method	74.4 (72.9–75.8)	77.3 (75.6–79.0)	68.8 (67.2–70.4)
IUD	26.6 (25.1–28.0)	21.4 (19.7–23.1)	27.0 (25.5–28.6)
Pill	10.7 (9.7–11.7)	12.5 (11.1–13.8)	11.3 (10.2–12.4)
Condom	21.6 (20.2–22.9)	26.5 (24.6–28.3)	26.2 (24.7–27.7)
Sterilization	2.5 (2.0–3.1)	2.3 (1.7–3.0)	1.3 (0.9–1.7)
Withdrawal	18.3 (17.0–19.6)	12.2 (10.9–13.6)	12.4 (11.3–13.5)
Periodic abstinence	9.1 (8.2–10.0)	14.0 (12.5–15.4)	6.5 (5.6–7.3)
Other	2.8 (2.2–3.3)	7.8 (6.7–8.9)	3.5 (2.9–4.1)
No method	25.6 (24.2–27.1)	21.8 (20.1–23.5)	31.2 (29.6–32.8)
No answer	--	0.9 (0.4–1.3)	--
Total number	4077	2032	4042

***Notes***: Respondents could indicate more than one method; therefore the sum does not equal 100%. Since we excluded pregnant women from our study, the results in this table do not coincide with those published in the official national MICS reports.

Numbers in the parentheses are 95% confidence intervals.

After standardization by age (the standard is the average population structure of the three samples), contraceptive prevalence constitutes 74.3 per sent in Belarus, 77.1 per sent in Russia, and 69.0 per sent in Ukraine.

***Source***: If not stated otherwise, the estimates presented in this and other tables in this section are based on the data from the Multiple Indicator Cluster Surveys (2005) for Belarus and Ukraine and on the data from the Generations and Gender Survey (2007) for Russia.

The Belarusian, Russian, and Ukrainian samples are rather similar in terms of the total contraceptive prevalence as well as of the composition of contraceptive methods. Two thirds of married or cohabiting women report to practice modern contraception. The most popular contraceptive methods in all the three countries are condom and intrauterine device (the first choice in Russia is condom, while in Belarus and Ukraine it is IUD) (Table 3). The third most common method in Russia is the calendar method, whereas in Belarus and Ukraine it is withdrawal (*coitus interruptus*); both these methods are considered ineffective. The pill is the fourth in the ranking: it is used by 11 per cent of women in Belarus and Ukraine and by 12.5 per cent in Russia. These results are different from official statistics (Figure S2), because official data on the use of pills count in all women having visited a doctor in a given year, while we restrict our sample to women in a union (marital or non-marital) only. The use of pills is notably lower in our studied countries than in other developed countries (e.g., in Western Europe it is 46 per cent on average [36]). Other types of hormonal contraception (injections, implants) are very rarely used in the three countries – by less than 0.5 per cent of women in union.

The world's most popular method of contraception – sterilization – has not gained high popularity in Belarus, Russia or Ukraine. According to the survey data, only 2.5 per cent of female respondents reported to have undergone sterilization procedure in Belarus, 2.3 per cent – in Russia, and 1.3 per cent – in Ukraine. Cases of male sterilization are extremely rare. The analysis of the Russian GGS data suggests that not all female respondents might have correctly understood the question concerning this method. There are grounds for suspecting that the prevalence of contraceptive sterilization might actually be even lower than shown by the survey results [37].

About one fifth of any method users in the three countries reported the simultaneous use of several contraceptive methods. Some combinations of methods indicated by one and the same woman, such as the simultaneous use of pills and implants or IUDs and pills, or IUDs and sterilization, make no sense. The highest proportion of women reporting combined use of four or more methods is found in Russia (1.3%); this proportion in Ukraine is 0.9%, and 0.5% in Belarus. The reporting of the use of multiple methods may reflect frequent migration from one method to another that reduces method effectiveness [38].

Table 4 shows the distribution of women by the most effective method currently used, i.e. if the respondent indicated more than one method, only the most effective one was considered. In defining method effectiveness, we relied on the ranking by Trussell [39]. It is important to note that this adjustment slightly changes the composition of methods. Since modern and traditional contraceptive methods are often used in a combination, it certainly increases the proportion of more efficient modern methods and correspondingly reduces the proportion of traditional methods. The analysis shows that the most commonly used methods in the three countries are intrauterine devices and condoms: almost half of women of reproductive age who have a partner use either one or the other method. The share of IUDs in Belarus and Ukraine is 27 per cent, while in Russia it makes 21 per cent. The respective figures for condom use are 18 per cent in Belarus, 21 per cent in Ukraine, and 23 per cent in Russia. According to Trussell's ranking where IUD is considered a more effective method than condom, the composition of methods typical for Russia is less effective.

**Table 4:** Distribution of women aged 15 to 49 who have a partner by the most effective contraceptive method out of those currently used, in per cent.

	Belarus, 2005	Russia, 2007	Ukraine, 2005
Any method	74.4 (72.9–75.8)	77.3 (75.6–79.0)	68.8 (67.2–70.4)
IUD	26.6 (25.1–28.0)	21.4 (19.7–23.1)	27.0 (25.5–28.5)
Condom	17.9 (16.7–19.2)	23.4 (21.6–25.1)	21.3 (19.9–22.7)
Pill	10.4 (9.4–11.4)	12.0 (10.6–13.3)	10.3 (9.3–11.3)
Sterilization	2.5 (2.0–3.1)	2.3 (1.7–3.0)	1.3 (0.9–1.7)
Periodic abstinence	5.7 (4.9–6.4)	7.6 (6.5–8.7)	2.9 (2.4–3.5)
Withdrawal	9.8 (8.9–10.8)	5.7 (4.8–6.7)	4.4 (3.7–5.1)
Other	1.5 (1.1–1.9)	4.8 (3.9–5.7)	1.5 (1.1–1.9)
Modern method (including in combination with traditional one)	57.9 (56.3–59.5)	60.8 (58.8–62.9)	61.0 (59.3–62.6)
Traditional method only	16.5 (15.3–17.7)	16.5 (14.9–18.0)	7.8 (6.9–8.7)
No method	25.6 (24.2–27.1)	21.8 (20.1–23.5)	31.2 (29.6–32.8)
No answer	--	0.9 (0.4–1.3)	--
Total	100.0	100.0	100.0

***Notes***: Method effectiveness is evaluated using Trussell's ranking [39].

Numbers in parentheses are 95% confidence intervals.

The proportion of couples using only traditional methods of contraception in Belarusian and Russian samples constitutes 16.5 per cent; this proportion is much higher than what is reported in Western European countries. In Ukraine, it is twice as low, about 8 per cent only, but in the 1990s, the proportion of users of traditional contraceptive methods was substantially higher in Ukraine [19], [40]. Moreover, Ukraine has the largest proportion of non-users among the three countries. Unfortunately, the surveys did not include questions about reasons for not using any contraception.

The analysis shows that the pattern of contraceptive behaviour considerably varies depending on the age of women (Figure S3).Young women are more likely to use condom or pills; IUD is more common among women aged 30 to 44; and after the age 40, there is a significant proportion of women who tend to rely on traditional methods (the latter is less pronounced in Ukraine though).

In addition, we tried to estimate *the unmet need for family planning*, that is, to identify the proportion of couples who were fertile, did not wish to have a child in the near future, and did not practice any means of pregnancy prevention. In order to calculate this indicator for Russia, we used the following GGS questions: “Do you intend to have a/another child during the next three years?”, “Is it possible for you, yourself, to have a/another baby, if you wanted it?”, and “Do you think it would be physically possible for your current partner/spouse to have a child of his own, if he wanted to?”. In the MICS, questions addressing these issues were slightly differently formulated. We focused on the questions allowing to distinguish respondents who could not get pregnant due to health-related reasons and those who wanted to get a child in the near future. For a better comparability with the Russian data, we selected those respondents, who were planning to have a child in 1–2 years or “soon”. In other words, we selected women, who were at the risk of conception and intentionally did not use any contraception. The Ukrainian MICS additionally contained a question: “What do you think, are you physically able to become pregnant now?”, which helped to produce a more accurate estimate for this country as compared with Belarus.

According to our estimation, the lowest unmet need for family planning, experienced by 13 per cent of couples, is in Russia. In Belarus, it is experienced by 16 per cent of couples, and in Ukraine – by 18 per cent. After standardization by age, the corresponding proportions remain almost unchanged. Irrespective of the observed differences, the unmet need in these countries is rather high by the world standards.

Although the wording of the questions and therefore the way of how the index of unmet need is estimated vary between the three countries, the unmet need in Russia is not likely to be higher than in Belarus and Ukraine. This brings us to a paradox – along with the highest level of abortions, Russia does not exhibit the highest unmet need for family planning.

Table 5 reports the results from binary logistic regression, where the dependent variable is use of any modern contraceptive method. A pooled dataset combining all the three samples is used. Model 1 presents the effect of each variable separately, adjusted for age only. Model 2 controls for all the variables, including the age.

**Table 5:** Odds ratios and 95% confidence intervals from logistic regression linking use of modern contraceptives to selected determinants.

	Model 1	Model 2
*Country*		
Belarus	1 (ref.)	1 (ref.)
Russia	1.16* (1.03–1.32)	1.16* (1.02–1.33)
Ukraine	0.86** (0.77–0.95)	0.82 (0.74–0.92)
*Number of children ever born*		
0	1 (ref.)	1 (ref.)
1	1.03 (0.75–1.43)	1.11 (0.79–1.55)
2	0.91 (0.66–1.26)	1.08 (0.77–1.53)
3+	0.70* (0.49–0.99)	0.94 (0.65–1.35)
*Place of residence*		
Rural	1 (ref.)	1 (ref.)
Urban	1.60*** (1.45–1.77)	1.51*** (1.36–1.67)
Capital city	1.50*** (1.27–1.77)	1.33** (1.12–1.58)
*Education*		
Lower than higher education	1 (ref.)	1 (ref.)
Higher education	1.30*** (1.18–144)	1.23*** (1.11–1.37)
*Type of union*		
Marriage	1 (ref.)	1 (ref.)
Cohabitation	0.95 (0.80–1.12)	0.88 (0.74–1.05)

*** p<0.001,

** p<0.01,

* p<0.05.

Women in union who do not want to have children in the next three years and are physically able to become pregnant.

The logistic regression shows that women in Russia are more likely to use modern contraceptives than women in Belarus or Ukraine. As expected, the odds of modern contraception use decrease after age 40 (not shown in the table; see Figure S3). The number of children does not appear to have a significant effect. Residence in rural area predicts lower use of modern contraception. Use of modern contraception is significantly higher for women with higher education. A significant association between contraceptive use and the level of education was also found in other studies on Russia [41], [42]. Finally, the type of union does not show any significant effect on the dependent variable.

## The Role of Family Planning Policies

Governmental policies play an important role in shaping contraceptive practices as well as achieving the shift from abortion to contraception being used for family planning purposes. Such policies promote the ideology of so-called planned parenthood, which assumes that couples and individuals make conscious efforts to control the number and spacing of births to make sure that all pregnancies are intended and all children are wanted. Regarding governmental policies, Belarus, Russia, and Ukraine considerably differ.

Ukraine pursues the most favourable and the most consistent with the above ideology policies. The development of national-level family planning programs began in the 1990s. In 1995, the Cabinet of Ministers approved the national target program named “Family Planning” (No. 736, September 13, 1995), outlining the action plan for the period until 2000. Further, in 2001, a Presidential Decree (No. 203/2001, March 26, 2011) was issued, which established the national program “Reproductive Health 2001–2005”. Finally, in 2006, the Cabinet of Ministers set the program “Reproductive Health of the Nation” for the period until 2015 (No. 1849, December 27, 2006). The implementation of these programs involved establishing a network of centres and clinics providing reproductive health and family planning services as well as setting up youth-friendly health facilities. These programs were successful in promoting responsible sexual behaviour and raising awareness and knowledge of modern contraceptive methods [43]. The Ukrainian Government subsidizes supply of modern contraceptives to vulnerable population groups, including economically disadvantaged women, women with serious heath problems, and young people. There are special budget allocations for these expenditures [31].

Although Belarus demonstrates the best achievements in reducing abortion incidence, no special programs that would promote family planning were initiated there. Reproductive health, sexual education, and other related issues were addressed in more general documents such as the national program “Women of the Republic of Belarus” or “The National Plan of Action for the Advancement of Women 1996–2000”. The closest to the subject of family planning is “The National Program of Planned Pregnancy and Prevention of Miscarriage, 2008–2010”, adopted in Belarus in 2008 (Order of the Ministry of Health of the Republic of Belarus No.42, January 23, 2008). This program aims at promoting use of and improving access to a wider variety of contraceptives; providing free contraception to women and adolescents from socially disadvantaged families and also those with medical contraindications to pregnancy; as well as establishing youth-friendly health facilities, which would include pregnancy and STD prevention counselling.

Russia was changing its stance in relation to family planning over time. In the early 1990s, the wave of democratic reforms taking place in Russia helped to adopt the federal target program “Family Planning”. In 1994, this program gained the support of the President of Russia (Decree No. 1696, August 18, 1994) and was integrated into the program called “Children of Russia”. The program “Family Planning” was designed to fundamentally change societal attitudes towards human reproductive rights and to create conditions for their realization. For the implementation of this program, the Russian Ministry of Health established a large network of centres for family planning and reproduction. Many professionals attended special advanced courses and received extensive training in related areas. A great deal of effort was expended to improve sexual culture of the Russian population; expert groups developed programs of sexual education. These activities encountered a strong resistance from some groups of the society. The Russian State Duma (formed mainly by communists at that time) supported the campaign against the program, and its funding was excluded from the state budget in 1998. Programs of sexual education were never introduced in schools.

The official position of the Government of Russia on having the right to family planning and reproductive choice as well as to safe, comfortable, and joyful sexual life has always been and remains ambiguous. Two decades ago, the segments of society holding traditionalist and fundamentalist views were either invisible or even non-existent. Today, they constitute an influential social force, which stirs up negative associations with and agitates against family planning. The belief in the myth that birth control is synonymous to low fertility and that broader access to family planning services inevitably leads to fertility reduction has become rather widespread. This myth has not only become part of folk common-sense, but also has successfully penetrated the level of decision makers. The resumption of a program, similar to “Family planning”, is hardly possible given the current pronatalist course proclaimed by the Government of Russia. On the contrary, many of the centres for family planning and reproduction created in the 1990s are gradually being closed due to lack of funding. Furthermore, by spreading fake information about abortion and its consequences for health as well as arguing about its moral unacceptability, the Government seems to be moving towards the decision that would restrict access to abortion [44], [45]. At the same time, the promotion of alternatives to abortion, i.e. contraception, is very limited.

The Orthodox Church agitates extensively against advances in reproductive health and rights, and this is typical of all the three countries under study. However, in Russia, the Orthodox Church seems to exert an especially strong influence: it successfully penetrated the public health decision-making process. The following is a quotation from the address by the Minister of Health and Social Development to participants of the second All-Russian Congress of Russian Orthodox doctors: “One of the important moments, where the role of the Church is especially significant, is the protection of family traditions and values and the prevention of and the reduction of abortions. We need to further pursue the campaign notifying about the harm caused to health by abortion, to inform people, particularly the youth, about potential complications, to talk about the psychological impact of abortion on women, and to create a proper mental attitude to motherhood” [46]. Moreover, the Foundation for Socio-Cultural Initiatives, headed by the First Lady of Russia, conducts an annual campaign called “Give me life!” aiming at protecting unborn children; it is promoted as a week against abortion [47]. All these trends do not contribute to the improvement of contraceptive use in Russia.

## Discussion and Conclusions

The hypothesis that the differences in abortion observed between Russia on the one hand and Belarus and Ukraine on the other hand is a statistical artefact was not confirmed. Belarus, Russia, and Ukraine have liberal abortion laws and inter-country differences in the regulations defining performance of abortion are minor. There are no legal barriers to availability or use of modern contraceptives in all these countries.

The data collection systems, as far as abortion statistics is concerned, are similar in Belarus, Russia, and Ukraine. They share the same or at least similar advantages and deficiencies, and the produced data are therefore of comparable quality and completeness. This finding allows to conclude that the divergence observed in the post-Soviet abortion trends in these countries is a genuine phenomenon.

Official statistics on the use of pills and IUD explain to some extent why abortion incidence in Belarus and Ukraine is lower than that in Russia. This suggests that Belarusian and Ukrainian women who are eventually visiting medical facilities are more actively using the two modern methods compared to their Russian counterparts. It is possible that the medical systems in Belarus and Ukraine are more committed to promoting and explaining the modern contraceptive methods among women which could lead to more consistent and qualified use of these methods.

This result contradicts to our findings based on the sample surveys. After adjustment for covariates, the Russian sample experiences higher odds of modern contraception (although the Russian method mix is slightly less efficient). This finding is unexpected given the higher level of abortion in Russia. It is also surprising that low fertility and rapidly declining abortion rates in Belarus and Ukraine go together with the inefficient structure of contraceptive methods; this fact attracted attention of other researchers too [48].

The paradoxical combination of higher contraceptive prevalence and higher abortion rate in Russia than in Ukraine was also noticed in the results of the 1999 surveys [49]. It is possible that Russian surveys exacerbate contraceptive prevalence due to propensity of Russian women to embellish their situation, sincerely confusing an intention to use or irregular use of a method with its proper use. However, Belarusian and Ukrainian surveys could face the same problem.

Thus, the survey data did not give an explanation of why Belarus and Ukraine experience greater progress in reducing abortion than Russia.

In our opinion, the main source of the inter-country discrepancies lies in contraceptive behavior itself, in adequacy of contraceptive knowledge and practices. In other words, it is important that women who use modern contraception do it in a proper way. It might be that higher contraceptive culture in Belarus and Ukraine in comparison to Russia leads to inter-country differences in rates of contraceptive failure. This explanation agrees with possible role of medical professionals that could underlie the growing gap between Russia and the two other Slavic countries in the use of pills and IUD.

The proportion of couples using traditional methods of contraception in these three countries is higher than what is reported in Western European countries. It is noteworthy that effectiveness of traditional methods can be very high: in the case of consistent and correct use, the failure rate is only 4–5 per cent [39]. The already mentioned Reproductive Health Surveys carried out in 1999 recorded higher failure rates in Russia than in Ukraine. Overall 12-month contraceptive failure rates were 12 per cent in Russia and 9 per cent in Ukraine, including 8 and 6 per cent respectively for pills, 23 and 16 per cent for periodic abstinence, 17 and 12 per cent for withdrawal [49]. In order to better understand the existing differences in the quality of contraceptive use, a survey should have other than the MICS/GGS design.

Differences in the state-implemented family planning policies in the three countries could also contribute to the explanation of the higher abortion rate in Russia. The Russian official (on the governmental level) stance on family planning has changed in the recent years to a highly traditional and pronatalist one. The myth that birth control is synonymous to low fertility and that better access to family planning services inevitably leads to fertility reduction is very strong in Russia. Such an extreme turn in the governmental position regarding family planning is not observed in Belarus or Ukraine.

The way in which information about contraception is obtained plays a significant role in efficiency of its use. The quality of information obtained from the Internet and other types of mass media, friends and acquaintances significantly differs from professional advice. Lack of medical services, sexuality education, and family planning promotion can result in inefficient contraceptive use and increase in the risk of unwanted pregnancy.

The Orthodox Church extensively campaigns against advances in reproductive health and rights. This campaign covers all the three countries. However, in Russia the church successfully penetrated the decision making process in public health, and its overall influence on the Russian government is particularly strong.

The latest developments related to family planning in Russia, which involve introduction of further legal restrictions in access to abortion, confirmed our guesstimates about the direction of the Russian governmental policies. We do not analyze these legal changes in this paper, but if our reasoning is correct, they will bring about a further growth in the abortion gap between the countries and may even terminate the ongoing decrease of abortion indicators in Russia.

## Supporting Information

Figure S1 **Rates of decline in abortion in Belarus, Russia, and Ukraine, per 1000 women aged 15–49. 1990 = 100%.**
(PNG)

Figure S2 **Women aged 15–49 using IUD and hormonal contraception (pills), in per cent. MoHs data.**
(PNG)

Figure S3 **Age patterns of contraceptive use of women with a partner by method, in per cent.** Source: Multiple Indicator Cluster Surveys (2005) for Belarus and Ukraine and the Generations and Gender Survey (2007) for Russia.
(TIFF)
