# Supplementary figures and images for: Divergent Trends in Abortion and Birth Control Practices in Belarus, Russia and Ukraine
Source: PLoS One. 2012 Nov 30;7(11):e49986. doi: 10.1371/journal.pone.0049986 (PMC3542819; doi:10.1371/journal.pone.0049986)

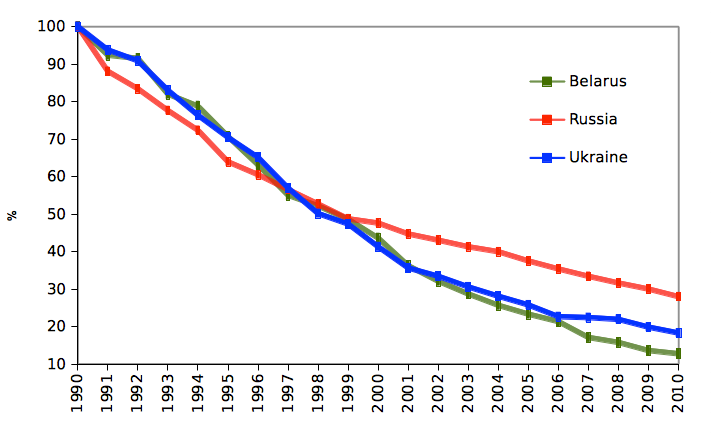

Supplement: Figure S1 — Rates of decline in abortion in Belarus, Russia, and Ukraine, per 1000 women aged 15–49. 1990 = 100%. (PNG) [file pone.0049986.s001.png]

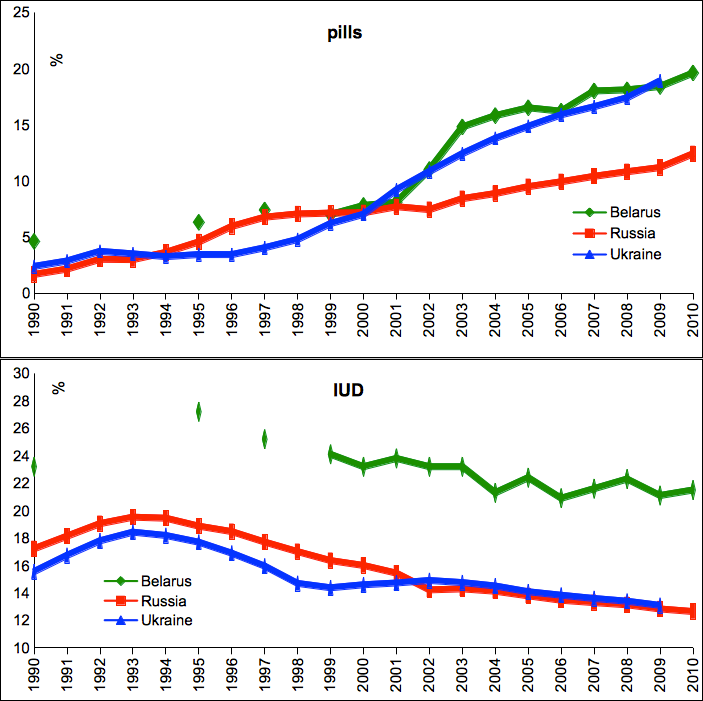

Supplement: Figure S2 — Women aged 15–49 using IUD and hormonal contraception (pills), in per cent. MoHs data. (PNG) [file pone.0049986.s002.png]

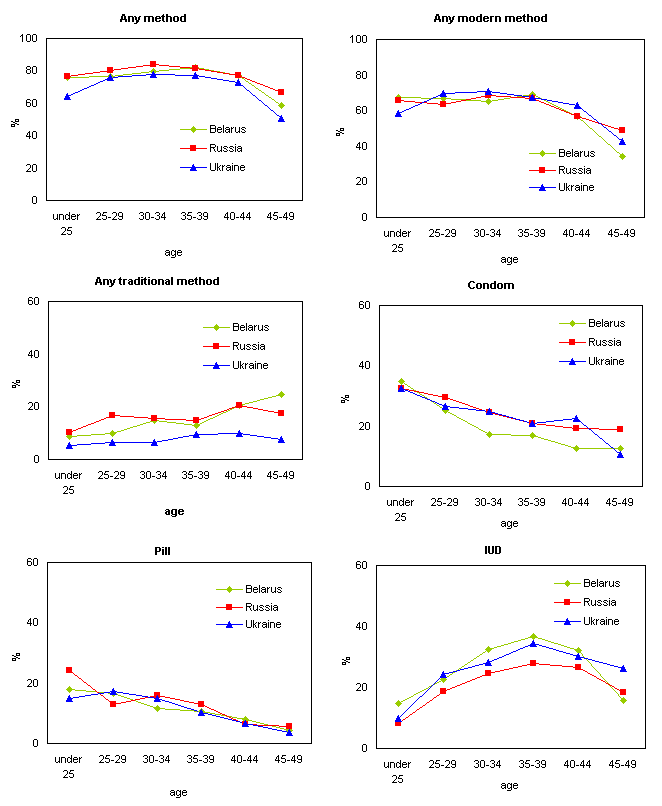

Supplement: Figure S3 — Age patterns of contraceptive use of women with a partner by method, in per cent. Source: Multiple Indicator Cluster Surveys (2005) for Belarus and Ukraine and the Generations and Gender Survey (2007) for Russia. (TIFF) [file pone.0049986.s003.tif]
